# MicroRNA in Aging: From Discovery to Biology

**DOI:** 10.2174/138920212803251436

**Published:** 2012-11

**Authors:** Hwa Jin Jung, Yousin Suh

**Affiliations:** 1 Departments of Genetics and Medicine, Albert Einstein College of Medicine, Bronx, USA; 2 Aging Research Institute, Guangdong Medical College, Dongguan, China

**Keywords:** Aging, Longevity, Gene regulatory network, High-throughput sequencing, microRNAs, Target validation.

## Abstract

MicroRNAs (miRNAs) are small non-coding RNA molecules that negatively regulate gene expression of their targets at the post-transcriptional levels. A single miRNA can target up to several hundred mRNAs, thus capable of significantly altering gene expression regulatory networks. In-depth study and characterization of miRNAs has elucidated their critical functions in development, homeostasis, and disease. A link between miRNAs and longevity has been demonstrated in C. elegans, implicating their role in regulation of lifespan and in the aging process. Recent years have witnessed unprecedented technological advances in studies of miRNAs, including ultra-high throughput sequencing technologies that allow comprehensive discovery of miRNAs and their targets. Here we review the latest experimental approaches from the perspective of understanding miRNA gene expression regulatory networks in aging. We provide a methodological work flow that can be employed to discover aging-related miRNAs and their targets, and to functionally validate their roles in aging. Finally, we review the links between miRNAs known to act in the conserved pathways of aging and major aging-related diseases. Taken together, we hope to provide a focused review to facilitate future endeavor of uncovering the functional role of miRNA in aging.

## 1 INTRODUCTION

MicroRNAs (miRNAs), first discovered in *C. elegans* [1], are small non-coding RNA species that regulate gene expression at the post-transcriptional level [2]. Mature miRNA are between 18-25 nucleotides (nt) in length and are initially transcribed as primary-miRNA (pri-miRNA) molecules which contain a characteristic stem loop structure. These non-coding RNAs undergo two processing steps [3] Fig. (**1**). The first step is the generation of stem-loop precursors (pre-miRNAs) of ~70nt in length by the enzyme Drosha in a micro-processing complex within the nucleus. The pre-miRNAs are then exported into the cytoplasm by exportin 5 and further processed into double stranded RNAs (miRNA-miRNA* duplex) by Dicer. The mature miRNA strand of this duplex is loaded into an Argonaute-containing miRNA-induced silencing complex (miRISC). In contrast, the complementary strand known as miRNA* (miRNA star) were thought to be degraded. However a growing body of work challenges the dogma that miRNA* is simply a non-functional byproduct of miRNA biogenesis, suggesting instead that miRNA* plays a significant role in cellular function and human disease [4, 5]. The mature miRNA within miRISC serves as a guide for recognizing target mRNAs by partial base-pairing.

The mature miRNA primarily targets the 3’ UTR of an mRNA strand based on sequence homology [6]. The nucleotides in the 2-7 position of the 5’ end of the mature miRNA comprise a “seed region”. Once an mRNA is targeted by a miRNA, its gene expression is down-regulated either by induction of mRNA degradation or blocking of translation by the miRNA, which occurs through conserved mechanisms [2, 7]. Perfect pairing of a miRNA with its target site supports endonucleolytic cleavage of the mRNA by Argonaute [8, 9]. Binding of the miRISC, which includes GW182 proteins, to 3’UTR target sequences has been shown to induce the recruitment of deadenylation factors that remove the poly(A) tail and make the mRNA susceptible to exonucleolytic degradation [10-15]. Although translation repression by miRNA occurs at the targeted mRNAs through inhibition of translation initiation or elongation [16-22], recent studies suggest that mRNA degradation is the primary mechanism by which miRNAs reduce protein output [23, 24]. 

One miRNA can target multiple mRNAs, and one mRNA can be targeted by multiple, distinct miRNAs; therefore miRNAs can significantly alter gene expression regulatory networks. The profound impact of miRNA on the gene regulatory networks has led to the in-depth study and characterization of miRNAs, elucidating their critical function in development, homeostasis, and disease such as cardiovascular disease [25] and neurodegenerative disease [26]. Thus far 1048 human miRNA sequences have been identified through cloning, sequencing, or computational analysis [27]. 

## 2 HIGH-THROUGHPUT SEQUENCING FOR DISCOVERY OF miRNAs AND TARGETs

The multitude of important roles played by miRNAs indicates that they are a critical genetic component of gene regulatory networks. However, quantification of miRNA has been technically challenging due to small size, low copy number, interference from other small RNAs, and contamination by degradation products of mRNAs or other RNA species. Until recently, the only known and computationally predicted miRNAs have been interrogated using hybridization-based array methods, an assay of limited value due to cross-hybridization, array content, and the inability to discover novel miRNAs. The increased availability and affordability of massively parallel sequencing offers a dramatically improved method to gain high-resolution views of miRNA expression [28, 29]. This technology has recently been utilized to profile expression of miRNAs in several species, including humans. Currently three commercial platforms for high-throughput sequencing are widely employed: Roche’s 454/FLX system, Illumina’s Genome Analyzer (formerly known as Solexa sequencing and succeeded by Illumina’s more recent model the HiSeq 2000) and ABI’s SOLiD. The choice of sequencing method often comes down to cost, read length and sequencing depth. Because miRNAs are in the range of approximately 18 to 30 nt and high sequencing depth is necessary to observe rare species, Illumina and SOLiD are currently the most cost-efficeint platforms for miRNAs sequencing studies.

Illumina uses a four-color, reversible terminator sequencing-by-synthesis technology to sequence one base at a time [28]. SOLiD uses 16 dinucleotide probes, each labeled with one of four fluorophores, to sequence by ligation two nucleotides of each clone at a time [30]. Sequencing cost has been further reduced by multiplex sequencing of indexed libraries, which allows sequencing of two or more samples in a single lane [31-39]. The number of samples for multiplexing varies depending upon the desired sequencing depth. By incorporating a unique sequence called a bar code or index into the 5’- or 3’-adapter of each library, or by adding the bar code during a PCR step after adapter ligation, the identity of each sample can be denoted. Multiplexing library preparation kits are now available for both Illumina and SOLiD. 

## 3 ANALYSIS OF HIGH-THROUGHPUT miRNA SEQUENCING DATA

There exist several analytical tools and databases that allow analysis of unprecedented amounts of miRNA-seq data from the high-throughput sequencing platforms for miRNA discovery and expression profiling as well as comparing miRNA profiles across a broad spectrum of species, tissues and diseases [40]. miRBase (http://www.mirbase.org/) is currently the repository for miRNAs. This database, which is updated regularly, stores information about the mature miRNA sequences, precursor sequences, map locations, and overlapping annotations, as well as predicted targets and a complete list of all publications that support each of the miRNA entries. An increasing number of species are included in every new miRBase version [41]. Other miRNA analysis platforms including miRDeep, miRNAkey, UEAsRNA toolkit, miRanalyzer, SeqBuster, DSAP, mirTools, E-miR and SigTerms are available for miRNA discovery and profiling and the identification of functional miRNA-mRNA pairs from deep sequencing [40]. miRecords is a resource for animal miRNA-target interactions, consisting of two components [42]; the *Validated Targets* component is a large, high-quality database of experimentally validated miRNA targets resulting from meticulous literature curation and the *Predicted Targets* component of miRecords is an integration of predicted miRNA targets produced by 11 established miRNA target prediction programs.

Recently GOmir has been developed for the target prediction and ontology clustering, which consists of two different components, JTarget and TAGGO [43]. JTarget combines the data from four different prediction databases (TargetScan, miRanda, RNAhybrid and PicTar) and also from the experimental database TarBase [44]. TAGGO provides detailed assignments from Gene Ontology (GO) resources to gene products. The expression patterns of miRNAs across tissues can be obtained from NCBI Gene Expression Omnibus GEO, miRGator [45], and microRNA.org. Databases such as miRSigDB [46] and miRGator allow high-level integration to understand how miRNAs expressed in a given sample relate to their putative targets in the context of signaling pathways. Furthermore, miRSigDB, miRGator, and recently published microRNA Expression and Sequence Analysis Database (mESAdb) permit miRNA expression and target gene expression to be linked to human diseases [47]. 

## 4 IDENTIFICATION OF FUNCTIONAL miRNA TARGETS

One of the challenges in the emerging field of miRNA biology is the identification of functional miRNA targets. A given miRNA may have multiple (up to several hundred) predicted gene targets, and ~60% of mRNAs have predicted binding sites for several miRNAs in their 3’ UTRs. But since miRNA regulation of an mRNA requires only a short (eight-nucleotide or fewer) match in their sequences, it has proved almost impossible to definitively determine which among many predicted mRNA binding sites is the *in vivo* target for each miRNA. Recently, several methods have been developed to identify molecular targets of miRNAs based on crosslinking approaches. 

### A High-Throughput Sequencing of RNA Isolated by Crosslinking Immunoprecipitation (HITS-CLIP)

High-throughput sequencing of RNA isolated by crosslinking immunoprecipitation (HITS-CLIP), is a genome-wide method to identify functional protein–RNA interaction sites [48]. This method utilizes ultraviolet (UV)-induced covalent crosslinking to stabilize RNA binding to its associated RNA-binding proteins, thereby enhancing the ability to capture more transient miRNA-mRNA interactions, prior to immunoprecipitation with antibodies for the protein. Deep sequencing of bound RNAs then allows comprehensive identification of functional interaction targets. HITS-CLIP can be used to covalently crosslink native Argonaute (Ago) protein-RNA complexes, producing two simultaneous datasets, Ago-miRNA and Ago-mRNA binding sites, that together allow identification of miRNA-target mRNA interaction sites through bioinformatic analysis [48]. Briefly, biological samples are irradiated with UV light (245 nm) and crosslinked samples are then immunoprecipitated with anti-AGO antibodies. Mild RNA digestion releases RNA flanking fragments that are not in direct contact with AGO. After stringent washing, only those miRNA and mRNA fragments directly crosslinked to AGO in RISC are preserved. These RNA tags are then released and deep-sequenced using massively parallel sequencing technology Fig. (**2**). Approximate miRNA binding sites on captured mRNA fragments are identified through the analysis and have been demonstrated to be enriched in miRNA target sequences [48]. HITS-CLIP of the RNA binding protein AGO has been applied to identify targets of miRNAs in mouse brain [49], and subsequently in *C. elegans *[50] and embryonic stem cells [51]. Recently, improved bioinformatics was applied to AGO HITS-CLIP, enabling identification of binding sites with single nucleotide resolution [52].

### B Photoactivatable-Ribonucleoside-Enhanced Crosslinking and Immunoprecipitation (PAR-CLIP)

PAR-CLIP, a modification of HITS-CLIP, utilizes photoactivatable ribonucleosides to enhance crosslinking efficiency and fold-recovery of RNA following wash steps [53]. The method relies on the incorporation of photoreactive ribonucleoside analogs, such as 4-thiouridine (4-SU) and 6-thioguanosine (6-SG), into nascent RNA transcripts by living cells. Irradiation of the cells by UV light (365 nm) induces efficient crosslinking of photoreactive nucleoside-labeled cellular RNAs to interacting RNA binding proteins. Crosslink formation between these modified ribonucleosides and RNA-binding proteins induces a high rate of modified T to C conversions during the reverse transcription process used prior to deep sequencing Fig. (**2**). Therefore, by analyzing locations of T to C conversions, the exact site of crosslinks can be determined, which greatly aids the process of identifying the miRNA target site on targeted transcripts. Hafner *et al.* used the epitope tagged Argonaute family members (AGO 1-4) expressed in HEK293T cells [53]. This study demonstrated that the most significantly enriched 7-mer motifs identified in co-immunoprecipitated RNA corresponded to the seed sequences of the most abundant miRNAs, which were generally positioned 1-2 nt downstream of the predominant cross-linking site. This implies that the site of crosslinking lies near the AGO-miRNA-mRNA complex and shows the ability for using T-C transitions to identify more specific sequenced DNA on miRNA:mRNA interaction sites [53].

These crosslinking strategies provide genome-wide data sets of endogenous miRNA targets and, potentially, their direct binding sites [54]*.* However, the detection of an mRNA bound by miRISC alone does not guarantee that it is actually being regulated, nor does it reveal the potential mechanism of control. HITS-CLIP and PAR-CLIP experiments may also reflect a selection bias for strongly interacting microRNA-ribonucleoprotein complexes [48], suggesting that they likely under-represent all miRNA target sites [48].

## 5 miRNA IN AGING

The potential of miRNAs to modulate aging in model organisms has recently attracted the interest of the molecular genetics community [55]. The importance of miRNAs in development has been firmly established, and increasingly, studies are linking altered miRNA function to a range of disease mechanisms [56]. Based on this research, there is every reason to believe that miRNAs play a major role in modulating life span and the aging process; indeed, support for this assertion has emerged from studies of model organisms as described below. 

### A miRNAs That Modulate Lifespan in Animal Models

Multiple miRNAs have been shown to regulate lifespan of *C. elegans* both positively and negatively [57-59], adding weight to the hypothesis that this gene class may contribute to robustness required for maintenance of healthy lifespan [60]. For example, the miRNA *lin-4* and its target *lin-14* control lifespan post-developmentally [57]; loss of function mutation of *lin-4* miRNA shortened life span and accelerated tissue aging, whereas knock-down of its target *lin-14* extends life span. Conversely, overexpression of *lin-4* extended lifespan by suppressing the target gene, *lin-14*, or lin-14 gain of function mutation, which lacks the *lin-4* binding sites in the *lin-14* 3’UTR leads to decreased longevity. Interestingly, knockdown of *lin-14 *only during adulthood is sufficient to increase longevity and suppress the *lin-4 *short-lived phenotype, indicating that these genes function in adulthood to modulate aging processes. In addition to *lin-4*, several other miRNAs that modulate longevity have recently been characterized and these miRNAs do not affect the developmental progression of *C. elegans*. *mir-71*, *-238 *and *-246 *mutants display a significantly shorter lifespan than those of wild-type animals, and over expressing miR-71 or miR-246 increases lifespan, indicating that these miRNAs function to promote longevity. Conversely, *mir-239 *mutants exhibit an increase in lifespan compared with that of wild-type animals, and miR-239 over expression produces the opposite effect, demonstrating that miR-239 antagonizes longevity [61]. Furthermore, expression patterns of these lifespan modulating miRNAs can be a predictor of lifespan [59]. Other miRNAs, including *let-7* and the muscle miRNA *miR-1*, have been described as potential modulators of age-related decline [62]. Recently, Kato *et al.* have shown that an adult-specific knockdown of *alg-1*, a *C.elegans* Argonaute gene, results in a significantly shorter lifespan compared with that of wild-type animals [63]. This indicates that a large-scale perturbation of miRNA maturation and function affects longevity. Since a significant number of miRNAs are evolutionarily conserved [64, 65], regulation of longevity by miRNAs is expected in higher organisms. Indeed, specific miRNAs up regulated during aging differ significantly between the long-lived Ames dwarf mice and their wild-type counterparts [66], implicating the function of miRNAs in delayed aging.

### B Age-Related Changes in miRNA Expression in Animal Models

Recently, in an effort to link miRNAs with processes of aging, global miRNA analyses on various aged tissues or organs have been performed. These studies revealed that more than 50 of the ~200 miRNAs in *C.elegans* reported in the miRBase are differentially expressed during aging, and more than half of these have conserved sequences in humans [60, 67]. *miR-34* stands out in particular, as it was found to be upregulated during aging, as well as during the dauer stage and early dormancy [61, 63, 68].

In mouse models, there exist differences in miRNA expression between young and old organs and tissues. While no significant differences were evident upon comparison of lung tissue from adult and aged lung [69], comparison of the brain and liver tissues of 10-, 18-, 24-, and 33-month-old mice through the miRNA microarrays and global proteomic profiling revealed that deregulated miRNAs were shared between the aging brain and aging liver, as well as between brain- and liver-specific miRNAs during normal aging [70]. Bates *et al*. have reported specific miRNA profiles in the livers of Ames dwarf mice (which are well known for their remarkable delay in onset of aging) using miRNA microarrays [66]. The results indicate that key enzymes involved in biosynthetic pathways such as ornithine decarboxylase and spermidine synthase are suppressed by *miR-27a*, and that this feature may contribute to the extended lifespan of the Ames dwarf mouse [66].

In humans, a comparative profiling of genes and miRNAs expressed in newborn, young adult and aged human epididymides was reported [71]. Since tissue is comprised of multiple cell types, the differential gene expression among different types of cells may compromise the ability to compare results. Nonetheless, the authors found that the newborn epididymis expressed the fewest mRNAs but the largest number of miRNAs, whereas the adult and aged epididymides expressed the most mRNAs but the fewest miRNAs, demonstrating a negative correlation between mRNAs and miRNA during aging. More recently, Noren Hooten *et al*. [72] utilized mononuclear cells from peripheral blood to evaluate miRNA expression in young and old individuals and revealed that the majority of miRNAs decreased in abundance with age. Predicted targets of the age-related down-regulated miRNAs include PI3K, c-Kit and H2AX, which were found to be elevated with advancing age, supporting a possible role for these miRNAs in the aging process [72]. 

### C miRNAs Acting on the Conserved Pathways of Aging

The rate of aging and lifespan are regulated by multiple conserved pathways of aging. Altering the pathways controlling metabolism, endocrine signaling, nutrient sensing, and stress resistance has been shown to prolong lifespan from yeast to mammals [73, 74]. Interestingly, the longevity-modulating miRNAs discovered in *C. elegans* are shown to function through the insulin/IGF-1 signaling (IIS) pathway, one of the first and best characterized conserved pathways of aging [57-59]. Mutations that impair the function of the IIS pathway extend lifespan in *C. elegans* [75], *Drosophila* [76], and mice [77], and are also implicated in human longevity [78]. This pathway is activated by binding of ligand (IGF-1 or insulin) to its receptor, DAF-2 in *C. elegans*, leading to an intracellular signaling that antagonizes the activity of Forkhead/FOXO transcription factors. Reduced function mutations in IIS genes activate Forkhead/FOXO proteins, DAF-16 in *C. elegans*, that regulate expression of several hundred genes implicated in metabolism, stress resistance and antimicrobial defense [79, 80]. Genetic studies have demonstrated that the *lin-4* miRNA (a longevity promoting factor) and its target *lin-14* (a life span antagonizing factor) function in the same pathway as DAF-2 and DAF-16. It was suggested that DAF-2 and LIN-14 negatively regulate DAF-16 function in parallel, whereas DAF-16 represses *lin-4* forming a possible negative feedback regulatory loop. Several studies report that multiple miRNAs regulate the components of the IIS pathway, such as *miR-1*, *miR-320*, and *miR-206* targeting IGF-1 [25, 81], and *miR-216a*, *miR-217*, and *miR-21* targeting PTEN [82, 83].

There are number of reports that forge a link between target of rapamycin (TOR) pathway and miRNAs. TOR is a major amino-acid and nutrient sensor that stimulates growth and blocks rescue pathways such as autophagy in response to nutrient and growth factor cues [23, 24, 84]. TOR proteins are highly conserved from yeast to humans. Inhibition of TOR signaling by rapamycin or by chronic dietary restriction decreases translation through activation of the translational repressor eIF4EBP and downregulation of ribosomal S6 kinase (S6K) and increases autophagy [68]. Inhibition of the pathway has also been reported to increase lifespan in many species, from yeast to mice [85-89]. Overexpression of *miR-100* inhibits both mTOR mRNA and protein levels, although there is currently no evidence of direct binding [90]. *miR-30a* targets *beclin-1*, the mammalian homologue of the yeast *Atg6*, by binding to its 3’ UTR and inhibits activation of autophagy induced by rapamycin [91]. Still, experimental evidence for a modulation of life span by these miRNAs is lacking, as is analysis of miRNAs in the context of caloric restriction. 

Several miRNAs have been reported to regulate the expression of SIRT1, an ortholog of yeast Sir2 implicated in regulation of life span, stress resistance, and metabolism [92]. Sir2 and other related members of the sirtuin family are highly conserved from yeast to mammalian cells. The surtuin proteins are NAD+-dependent protein deacetylases that regulate the activity of many proteins involved in energy metabolism, inflammation, transcription, and cell survival [93]. *miR-217* expression is progressively increased during ageing in endothelial cells, and it can modulate SIRT1 expression through binding to the 3’UTR of SIRT1 mRNA [94]. *miR-34a*, a downstream target of p53, has also been found to target SIRT1 in mouse liver [95], indicating that there is a connection between *miR-34a* and the ageing signaling pathway. SIRT1 is also a direct target of *miR-199a* and *miR-132* and mediates the regulation of chemokine production [96] or HIF-1α function [97]. 

### D miRNAs in Age-Associated Diseases

Recently, some miRNAs that target conserved pathways of aging, including Insulin/IGF signaling (IIS), DAF-12 signaling and TOR signaling, have been linked to human aging-related disorders such as heart [98-108], muscle [109, 110], and neurodegenerative disease [111, 112] Fig. (**3**). *miR-1*, *miR-122* and *miR-375* target IIS and have been associated with heart disease. There is evidence that downregulation of *miR-1* is correlated with hypertrophic growth of heart in both mice and humans [103]. Plasma levels of *miR-122* and *miR-375* are decreased in patients that present with myocardial infarction [102]. *miR-21*, which is activated by TOR and NFκB in hepatocytes, is one of the most highly and consistently upregulated miRNAs during cardiac hypertrophy [99, 106-108]. Reconstitution of *miR-21* within an infarct zone reduces cell death and infarct size and ameliorates cardiac dysfunctionalthough the role of *miR-21* in heart through the TOR and NFkB pathway is not studied yet.

*miR-1* and *miR-206* regulate IIS [81, 110] and play have a role in skeletal muscle hypertrophy and atrophy [109, 110]. The expression of these miRNAs increased during development of human skeletal muscle, indicating that these miRNAs are also involved in the development of human skeletal muscle [113, 114] and myogenesis by targeting myogenic factors such as MEF2, serum response factor (SRF), and myostatin [115]. *miR-1* expression are decreased during skeletal muscle hypertrophy [116]. miRNA profiling in skeletal muscle identified *miR-206* as an up-regulated miRNA with age in mice [109]. *miR-206* can induce muscle hypertrophy and its increased expression with muscle atrophy in aging may indicate an adaptive, compensatory response to antagonize other catabolic signals [109]. The reason for the differential expression of these miRNAs in muscle disorders is still unclear and needs further investigation.

miRNAs also play key roles in controlling metabolic homeostasis and diseases [117]. *miR-21* expression is increased in the livers of rats fed high-fat diets and in human liver biopsies of obese patients with diminished *PTEN* expression, in line with the findings that *miR-21* is activated by an mTOR/NF-κB-dependent mechanism and inhibits *PTEN* by binding to its 3’UTR [118]. Aberrant up-regulation of *miR-21* expression by excessive circulating levels of fatty acids exemplify a novel regulatory mechanism by which fatty acids affect *PTEN* expression and trigger liver disorders [118]. In contrast, *let-7* inhibits adipogenic differentiation through the down-regulation of adipogenic factors [119-124]. The tumor suppressor roles of *let-7* are well studied in cancer biology, but *let-7* was recently also shown to be involved in the regulation of glucose metabolism [125]. This effect may, at least partially, be mediated by repression of insulin-like growth factor receptor 1 (*IGF1R*), insulin receptor (*INSR*) and *IRS2* [125].

Many studies have shown the alteration of miRNA expression in neurodegenerative diseases including Alzheimer’s disease and prion-induced neurodegenerative disease [126]. *let-7*, known to target DAF-12 signaling and regulate lifespan in worms [127], has been implicated in Alzheimer’s disease by its demonstrated genetic interactions with the homolog of amyloid precursor protein (APP), APP-like-1 (*apl-1*) in worms, suggesting that Aβ peptide formation is under miRNA control in organisms other than mammals [111, 112]. Recently, *miR-320* which is known to target IGF-1 and IGF-1R in rats [128] is found to be up-regulated in prion-induced neurodegenerative disease [129]. In summary, de-regulation of miRNAs acting on the conserved pathways of aging in a variety of aging-related disorders strengthens the notion that aging is a root cause of aging-related diseases.

## 6 CONCLUSION

The discovery of miRNAs points to an entirely new regulatory module to control biological processes. Recent studies are linking altered miRNA function to a range of aging-related diseases and processes of aging. The increased availability and affordability of massively parallel sequencing offers a dramatically improved method to gain a high-resolution view of miRNA expression. In addition, high-throughput technologies allow the identification and the validation of miRNA target genes, providing new approaches to identify miRNA regulatory networks in aging Fig. (**4**). Identification of miRNAs that modulate aging will provide important mechanistic insights into the molecular basis of aging.

## Figures and Tables

**Fig. (1):**
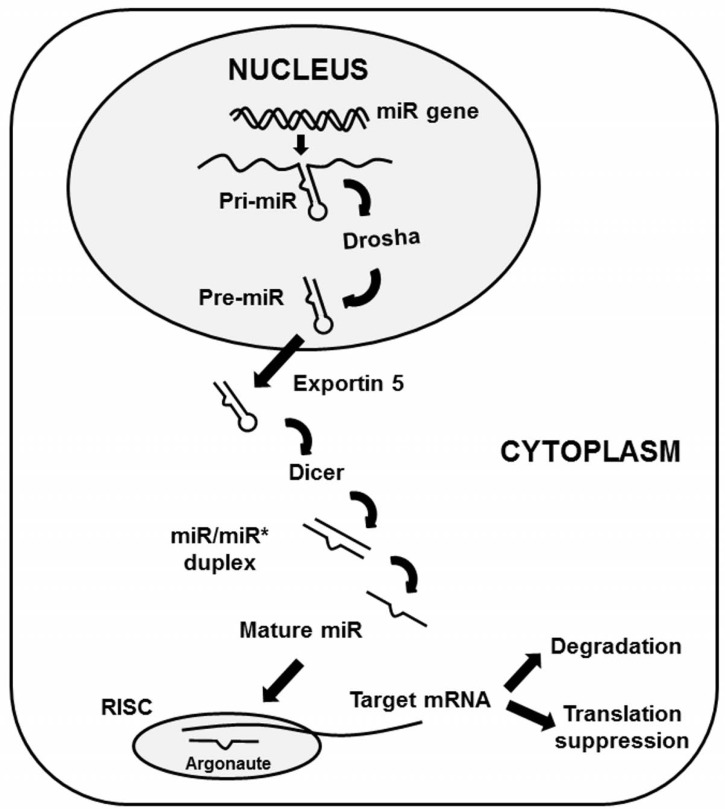
**Biogenesis of microRNA**. MicroRNA (miRNA) genes are typically transcribed into primary miRNA (pri-miRNA) transcripts that undergo processing by Drosha-containing complexes in animals. The unified hairpin precursor miRNAs (pre-miRNAs) are transported to the cytoplasm by exportin 5 (XPO5). The Dicer complex removes the loop region from pre-miRNAs, and one strand of the resulting duplex is bound by Argonaute to form a miRNA-induced silencing complex (miRISC). After multiple processing, mature miRNAs target complementary transcripts and downregulate gene expression by mRNA degradation or translational repression.

**Fig. (2):**
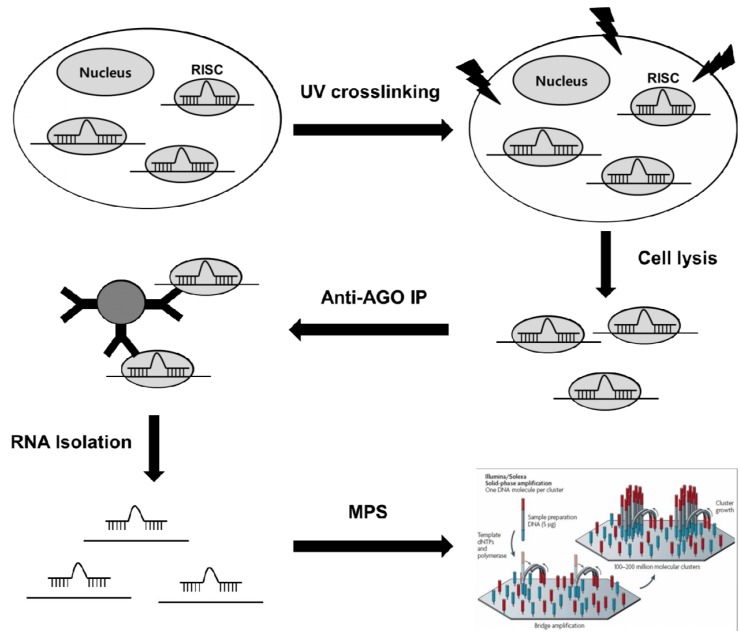
**High-throughput methods for identification of miRNA target genes**. HITS-CLIP and PAR-CLIP utilizes ultraviolet (UV)- induced covalent crosslinking to stabilize RNA-Argonaute (AGO) protein complexes in miRISC, thereby enhancing the ability to capture more transient miRNA-mRNA interactions, prior to immunoprecipitation (IP) with antibodies. Massively parallel sequencing (MPS) of bound RNAs then allows comprehensive identification of functional miRNA-target mRNA interaction sites.

**Fig. (3):**
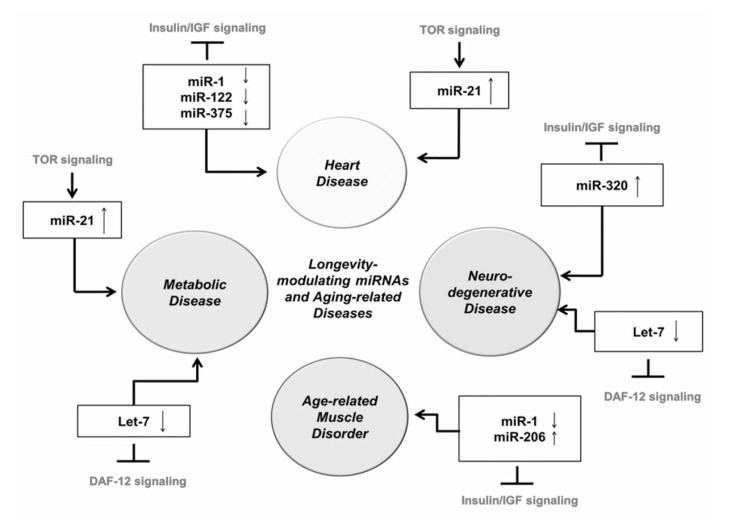
**MiRNAs involved in conserved pathways of aging and their role in age-related diseases in humans**. A schematic representation of miRNAs known to target genes involved in the conserved pathways of aging and their connections to age-related diseases.

**Fig. (4):**
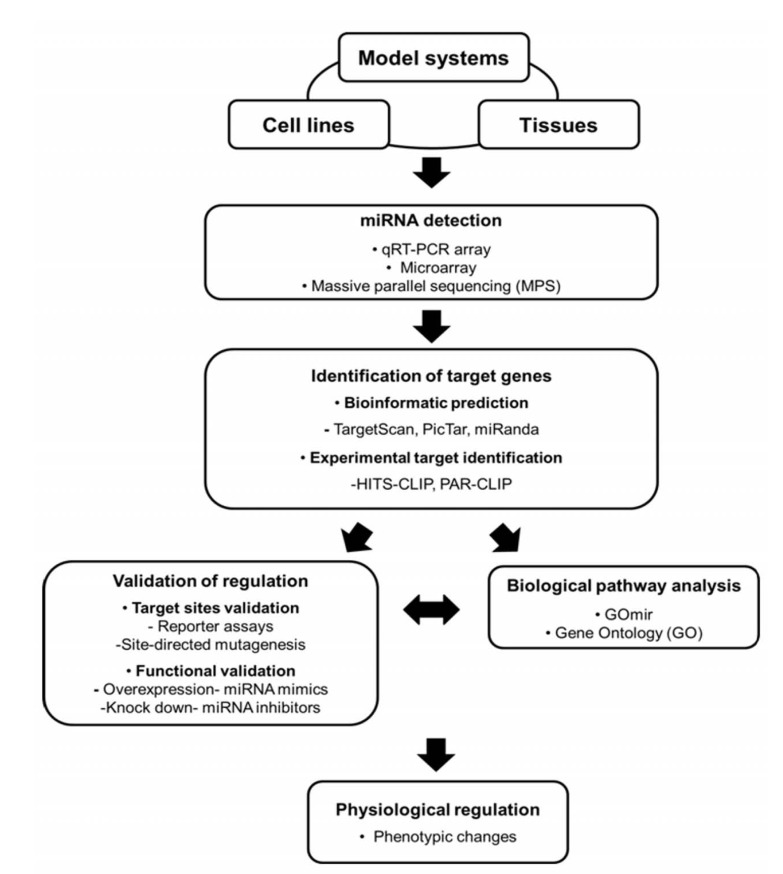
**Summary of methodological workflow for studying miRNA function**. The workflow summarizes the emerging high-throughput experimental approaches for the study of miRNA gene regulatory networks in aging. For miRNA discovery, high-throughput methods such as massively parallel sequencing or microarray can be utilized to identify differentially expressed miRNAs in a variety of aging models. Target sites of aging-related miRNAs are identified either by *in silico* analysis or experimental approaches such as HITS-CLIP and PAR-CLIP. Target sites are then validated by 3’ URT reporter assays or by assessing the anti-correlation between a miRNA and its target gene/protein levels in transfection experiments with miRNA minics, antagomir, or target protectors. Biological pathway analysis of target genes will provide insights into aging gene regulatory networks.
